# Mitigating psychological distress in healthcare workers as COVID-19 waves ensue: a repeated cross-sectional study from Jordan

**DOI:** 10.1186/s12960-022-00728-x

**Published:** 2022-04-11

**Authors:** Nour A. Obeidat, Yasmeen I. Dodin, Feras I. Hawari, Asma S. Albtoosh, Rasha M. Manasrah, Asem H. Mansour

**Affiliations:** 1 Cancer Control Office, King Hussein Cancer Center, Amman, 11941 Jordan; 2 Office of Minister of Health, Amman, Jordan; 3 Respiratory Division, Internal Medicine Department, University of Jordan, Amman, Jordan; 4 Director General Office, King Hussein Cancer Center, Amman, Jordan

**Keywords:** Psychological distress, COVID-19, Jordan, Health personnel, Mental health

## Abstract

**Background:**

Jordan has experienced several COVID-19 waves in the past 2 years. Cross-sectional studies have been conducted to evaluate distress in healthcare practitioners (HCPs), but there is limited evidence with regards to the impact of continuing pandemic waves on levels of distress in HCPs. We previously studied psychological distress in HCPs during the start of the pandemic (period 1, when cases were infrequent and the country was in lockdown), and demonstrated that HCPs were experiencing considerable stress, despite the country reporting low caseloads at the time. In this study, we sought to utilize the same methodology to reexamine levels of distress as COVID-19 peaked in the country and HCPs began managing large numbers of COVID-19 cases (period 2).

**Methods:**

A cross-sectional online survey utilizing a tool previously used during period 1 was completed by HCPs working in various settings. Demographic, professional and psychological factors such as distress, anxiety, depression, burnout, sleep issues, exhaustion, and fear were assessed; and coping strategies also were measured. Items in the tool were assessed for reliability and validity. A multivariable regression was used to identify factors that continued to impact distress during period 2.

**Results:**

Samples in both periods (*n* = 937, *n* = 876, respectively) were relatively comparable in demographic characteristics, but in period 2, a greater proportion of nurses and healthcare practitioners reported working in general hospitals. During the pandemic peak (period 2), 49.0% of HCPs reported high levels of distress (compared to 32% in period 1); anxiety and depression scores were approximately 21% higher in period 2; and 50.6% reported fatigue (compared to 34.3% in period 1). Variables significantly associated with greater distress in period 2 included experiencing burnout, experiencing sleep disturbances, being fatigued, having fatalistic fears, and having fears related to workload. Conversely, being male, reporting satisfaction at work, and using positive coping practices were associated with a significantly lower odds of being in distress.

**Conclusions:**

Between the two periods (early pandemic and first wave), COVID-19-related mental health continued to deteriorate among HCPs, highlighting the need to do more to support HCP front-liners facing COVID-19 surges.

**Supplementary Information:**

The online version contains supplementary material available at 10.1186/s12960-022-00728-x.

## Introduction

The severe outbreak of a novel coronavirus disease 2019 (COVID-19) has had devastating health consequences worldwide [1]. Jordan, a country located in the Middle East, only began to experience its first sharp wave of COVID-19 cases in October of 2020. Prior to this, and as the global community grappled with COVID-19, Jordan was spared due to stringent lockdowns and border closures imposed early in the global pandemic (as early as March, 2020). Jordan’s first wave occurred after restrictions were eventually eased and borders reopened in September of 2020. The first wave peaked towards the end of November, and began to recede by the end of 2020. A second sharp wave occurred in March of 2021, and at its peak, Jordan reported the highest number of new cases in the Eastern Mediterranean Region (57 666 new cases; 565.2 new cases per 100 000) [2]. By the summer of 2021, COVID-19 incidence rates had declined as vaccination rates continued to increase [3], but subsequent waves of COVID-19 infections have been declared since then [4, 5].

At the onset of the pandemic, much global focus was rightfully placed on understanding the impact of the outbreak on the psychological health of healthcare practitioners (HCPs). HCPs across all disciplines represent a vulnerable group, and have been subjected to greater health risks, stress, burnout, isolation, and heightened fear during the pandemic [6, 7]. A tremendous amount of evidence (largely in the form of cross-sectional studies and systematic reviews of these studies) has been generated to demonstrate COVID-19’s impact on HCPs’ psychological and mental health outcomes, such as depression, anxiety, burnout, fatigue and sleep disturbances [8–13].

In Jordan, the few studies examining HCPs’ psychological well-being during the pandemic have demonstrated similar effects: Jordanian HCPs have experienced considerable psychological distress, depression, anxiety, fatigue, sleep disturbances, and burnout [14–18]. However, these studies have varied in terms of their timing (relative to the COVID-19 waves that occurred in the country), mode of measurement of mental health outcomes, and overall findings. Furthermore, while useful in examining what was happening at a single point in time, the variability in measurement tools across these studies has limited the extent to which they can be compared, making it difficult to decipher the impact of different epidemiological stages of the pandemic on HCP-reported mental health. Such information is valuable to have, particularly for the purpose of providing insight about the challenges faced by HCPs’, and how they can be better supported and prepared in the event of future waves of COVID-19.

**Fig. 1:**
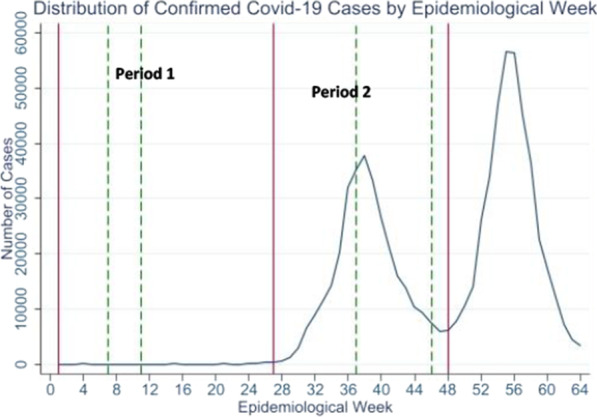
Caseloads reported in Jordan relative to the periods in which the two surveys were conducted

The aim of our study was—using a previously applied approach—to conduct a repeated cross-sectional study to re-evaluate Jordanian HCPs’ levels of fear, distress, anxiety, depression, sleep quality and fatigue, after experiencing a COVID-19 surge. Our first cross-sectional survey captured the early [low caseload] pandemic stage and generated valuable baseline information [16]. However, 6 months after the study, in the fall of 2020, COVID-19 cases and deaths dramatically escalated, resulting in about 320 207 new cases and 4233 deaths (compared to 2034 cases and 15 deaths during the early pandemic stage, Fig. 1) [19]. We hypothesized that, after experiencing a sharp COVID-19 wave, HCPs’ levels of fear, distress, anxiety, depression, sleep quality, and fatigue would further deteriorate despite already being predisposed (as demonstrated in our first cross-sectional study) to the possibility of COVID-19 spread. We also hypothesized that determinants of distress would remain consistent, and that even in high caseload settings, specific sociodemographic, attitudinal and occupational factors—namely, male gender, age, positive perceptions related to work, and low levels of fear regarding COVID-19, would continue to be significantly protective against psychological distress.

## Materials and methods

### Study design and sample

We used a similar approach to that taken in our first cross-sectional study [16]. Specifically, a second cross-sectional Arabic online survey (https://www.questionpro.com/) was distributed across key governmental and academic hospitals and in community pharmacies largely in the Central region of the country. Distribution channels were purposeful, targeting physicians, nurses, technicians, and pharmacists. Channels included email, text-messaging, and social media groups restricted to healthcare professionals potentially working in these key institutions. The second questionnaire was available from November 15, 2020 and until mid-January of 2021.

### Research questionnaire and study variables

The research instrument employed for the study has been previously described [16]. Briefly, in our first study, we were interested in capturing various constructs related to distress as well as occupational health but no single published tool captured the entirety of constructs we were interested in. To develop a final questionnaire of reasonable length, we employed various tools (e.g., short-form PROMIS measures and single-item measures such as the burn-out measure were used). We ensured face and content validity through a group of experts and medical staff involved in both research and COVID-19 screening and potential management, and subsequently (during data analysis) examined and confirmed the reliability of the items.

The instrument was composed of the following measures:

- Distress [20, 21]: distress in the past 30 days remained our primary outcome of interest. Distress was categorized into four levels, namely, no distress (score = 0), low distress (scores = 1 to 5), moderate distress (scores = 6 to 10), and high distress (scores = 11 to 24).
- Burnout (single-item, five-level measure) [22]: respondents who identified with the third level “I am definitely burning out and have one or more symptoms of burnout, such as physical and emotional exhaustion” or greater were considered as suffering from burnout.
- Patient-Reported Outcomes Measurement Information System (PROMIS) measures of anxiety and depression in the past 7 days [23, 24]: a cutoff of 11 (from a total score of 20) was used to identify at least moderate anxiety or depression [25, 26].
- PROMIS measures of sleep-related issues in the past 7 days [27, 28]: sleep issues were considered present if respondents reported trouble falling asleep or staying up at least half of the night “quite a bit” or “very much” in the past 7 days.
- PROMIS measures of fatigue in the past 7 days [29]: fatigue was operationalized as present if respondents reported feeling exhausted “quite a bit” or “very much” in the past 7 days.
- Sources of fear [30]: 19 five-point Likert scale fear items originally referring to severe acute respiratory syndrome (SARS) were adapted to refer to COVID-19; additional items pertaining to the local (Jordanian) context were further included in the first survey (e.g., fear of going to work; fears concerning financial stability) and in the second survey (fear of being quarantined at home or at other sites). Factor analysis also was conducted to examine the factor structure of the fear items and to reduce the number of variables for subsequent analyses. Six final factors were identified (Additional file 1).
- Workplace characteristics and perceptions about working environment [31]: ten items were included to measure level of agreement (on a five-point Likert scale) with statements concerning satisfaction at work, perceived communications and camaraderie at work, and feeling respected and appreciated.
- Availability of specific personal protective equipment [32]: availability of individual equipment was measured, and a summary variable (having access to a surgical or N95 mask, an eyeguard, gloves, a gown, and shoe covers) was generated (hereby referred to as “PPE availability”).
- A demographics and professional characteristics section.

In the second survey, two sections were added to gain further insight about HCPs’ experiences.

- Coping mechanisms: to gauge coping strategies used by HCPs, 15 potential strategies were explored. Coping strategies were adapted from other studies that were conducted in comparable situations [33–35]. For each strategy, HCPs were asked to rate the strategy from 0 to 3 (0: never used; 1: sometimes used; 2: often used; 3: always used). Factor analysis also was conducted to examine the factor structure of the coping strategies and their internal consistency. Three final factors were identified (Additional file 1).
- Degree of importance of select factors for HCPs’ work: this section consisted of 12 items. Nine items were adapted from previous studies [33, 34], and included financial compensation in the event of illness; recognition of efforts; availability of PPEs, vaccines, treatments, and psychological support services at work; and measures to control workload). Three items were included to reflect issues specific to Jordan that HCPs may have been experiencing (poor recognition of efforts from the public, poor representation from the media, and salary deductions). Factors were assessed using a four-point Likert scale (ranging from 0, “not important” to3, “most important”).

### Statistical analyses

Similar to our approach in the first survey, descriptive statistics were first conducted to characterize levels of distress, fear, anxiety, depression and burnout, and their correlation with one another; and to explore how distress varied across various demographic, professional and attitudinal (fear) characteristics. Chi-square tests, Pearson's correlations, *t* tests and ANOVA tests were run to explore potential bivariate associations between variables. Comparisons also were made between distress, anxiety, depression and burnout levels reported in the first and second surveys.

Specifically with regards to fear and coping strategies, factor analysis and data reduction were conducted for the purpose of reducing the multiple items in each construct into fewer summary variables (Additional file 1) [36]. In the factor analysis of fear, six factors emerged, which we used to summarize the 21 fear items: (i) fears related to respondents’ families; (ii) fears related to the respondent becoming infected; (iii) fatalistic fears about the virus being out of control and thoughts about death; (iv) quarantine fears; (v) fears related to nature of work; and (vi) monetary fears. In the factor analysis of coping, three factors emerged, which we used to summarize the 14 coping items: (i) coping using positive practices; (ii) coping by seeking COVID-related information and controlling risk of infection; and (iii) coping through denial, avoidance, crying, or negative reactions. Average scores were generated for each factor, and scores for both groups of factors (fear factors, coping factors) were subsequently utilized as covariates in the multivariable analysis. Further details are provided in Additional file 1.

A final multivariable analysis was conducted to identify significant factors that were associated with increased odds of being in a higher distress category. An ordinal logistic regression was used given the nature of the dependent variables (four levels of distress). The final model included significant variables at the bivariate level: basic demographic and professional characteristics, work-related experiences, measures of occupational health (e.g., experiencing sleep issues, exhaustion, or burnout), and the predicted scores of fear and coping factors. Model diagnostics were run to ensure that the multivariable model did not violate the proportional odds assumptions of ordinal logistic regression [37].

All analyses were conducted using STATA 16 [38–40]. We reported significance values using both a conventional cutoff of 0.05 and a conservative Bonferroni-adjusted cutoff value (due to concerns related to multiple comparisons).

## Results

### Characteristics of the sample

**Fig. 2:**
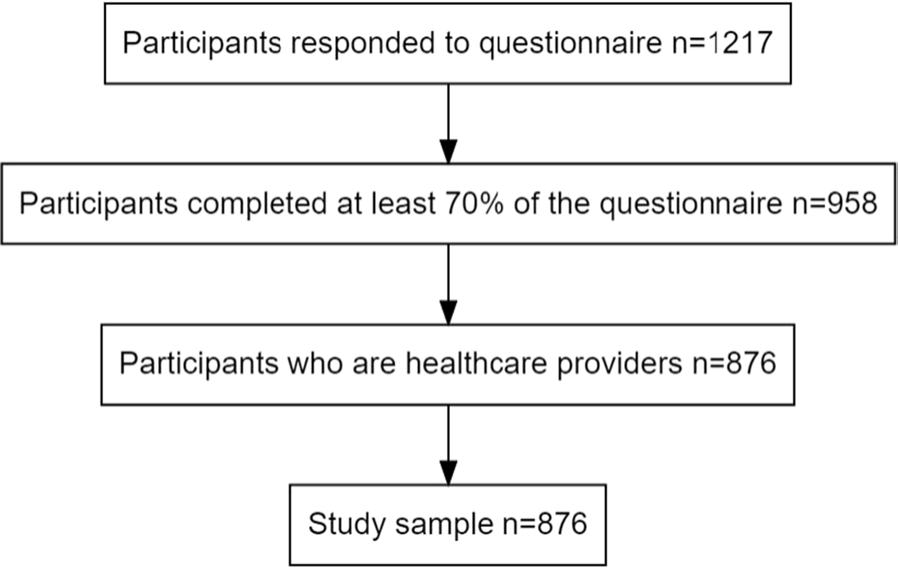
Study sample flow diagram

A total of 1217 subjects responded to the survey in period 2. After excluding surveys with substantial missing information and excluding employees in healthcare organizations that were not healthcare providers, our final analytic sample was composed of 876 HCPs (Fig. 2).

Approximately 60% (*n* = 511) of respondents were females, and the majority of subjects (79.9%, n = 700) were aged between 18 and 40 years. The largest proportion of HCPs consisted of nurses and technicians (70.7%, *n* = 619), while physicians and pharmacists comprised 20% (*n* = 175) and 9.4% (*n* = 82) of the sample, respectively. Approximately 61% (*n* = 528) of respondents worked in general hospitals, medical centers and private clinics; 37% (*n* = 320) worked in a specialized cancer center; and 3% (*n* = 22) worked in community pharmacies. One-fourth of the sample (25.5%, *n* = 241) had been infected with COVID.

Table 1 demonstrates the sociodemographic, professional and work characteristics of the sample in period 2, relative to the sample in period 1. The sample in period 2 was relatively comparable to that in period 1 in terms of age, gender distribution, educational status, and marital status (but less respondents in period 2 resided with young people). The sample in period 2 also had significantly more nurses than physicians and pharmacists, and a larger proportion of healthcare practitioners working in general (non-cancer) hospitals. With regards to the key outcome of interest, distress, and associated variables, the second sample exhibited significantly higher levels of distress, anxiety, depression, sleep disturbances, and fatigue. With the exception of gloves, availability of PPE was significantly greater in the second sample.

**Table 1:** Characteristics of sample of healthcare practitioners responding to second survey, relative to sample responding to first survey (column totals presented)

	Sample 1 (pre-COVID-19 wave)	Sample 2 (post-COVID-19 wave)	*p* value
General and demographic variables			
Completed the two surveys, n (%)	NA	310 (32.7%)	NA
Diagnosed with COVID, n (%)	NA	241 (25.5%)	NA
Sample size	937	876	NA
Date collected between (month/year)	4/20 to 5/20	11/20 to 1/21	NA
Age, mean in years (SD)	33.3 (7.9%)	33.2 (7.9%)	0.85
Male, n (%)	411 (43.9%)	365 (41.7%)	0.345
Live with spouse, n (%)	592 (63.7%)	550 (62.8%)	0.701
Have children, n (%)	528 (56.4%)	472 (53.9%)	0.291
Live with old people, n (%)	417 (44.5%)	418 (47.7%)	0.170
Live with young people, n (%)*	748 (79.8%)	650 (74.2%)	0.004
Education level			
Diploma or less, n (%)	113 (12.1%)	94 (10.7%)	0.501
Bachelor degree, n (%)	669 (71.4%)	623 (71.1%)	
Masters, PhD, n (%)	155 (16.5%)	159 (18.2%)	
Professional and workplace characteristics			
Occupation*^ψ^			
Nurses and technicians, n (%)	629 (68.3%)	619 (70.7%)	0.000
Physicians, n (%)	126 (13.7%)	175 (20.0%)	
Pharmacists, n (%)	166 (18.0%)	82 (9.4%)	
Type of institution (government or academic)*^ψ^			
Specialized hospital (cancer), n (%)	390 (41.6%)	320 (36.8%)	0.000
Non-cancer/general hospital/medical center/clinic, n (%)	427 (45.6%)	528 (60.7%)	
Community pharmacy, n (%)	115 (12.3%)	22 (2.5%)	
Mean years of experience in the field (standard deviation)	9.9 (7.7)	9.5 (7.4)	0.372
Site of work			
Hospital ICU & ER, RTU n (%)	264 (28.4%)	261 (30.4%)	0.640
Hospital medical departments, n (%)	543 (58.4%)	486 (56.6%)	
Other sites, n (%)	123 (13.3%)	111 (13.9%)	
COVID-related work characteristics			
Dealt with suspected or actual COVID patients in line of work (actual or suspected), n (%)*^ψ^	462 (49.3%)	819 (93.5%)	0.000
Work in a COVID-19 specialized ward, n (%)*^ψ^	148 (15.8%)	362 (41.3%)	0.000
Experienced a high workload during past 30 days, n (%)*^ψ^	315 (33.6%)	496 (56.6%)	0.000
Was satisfied at work, n (%)*	670 (71.7%)	577 (65.9%)	0.009
Agreed that co-workers could be relied on, n (%)	486 (52.0%)	451 (51.5%)	0.853
Agreed that peers could openly talk, n (%)	616 (65.9%)	560 (64.0%)	0.402
Agreed there were effective work safety measures, n (%)	540 (61.0%)	515 (58.8%)	0.355
Agreed that sufficient PPE training was given, n (%)	458 (51.7%)	466 (53.2%)	0.528
PPE availability			
Availability of full PPE, n (%)*^ψ^	302 (32.2%)	393 (44.9%)	0.000
Psychological health			
Distress*^ψ^			
None, n (%)	29 (3.1%)	13 (1.5%)	0.000
Low, n (%)	287 (30.6%)	160 (18.3%)	
Moderate, n (%)	321 (34.3%)	274 (31.3%)	
High, n (%)	300 (32.0%)	429 (49.0%)	
Reported at least one symptom of Burnout, n (%)*^ψ^	314 (33.5%)	411 (46.9%)	0.000
Anxiety, past 7 day raw score, mean (SD)* ^ψ^	9.2 (3.7)	11.1 (4.1)	0.000
Depression, past 7 day raw score, mean (SD)* ^ψ^	7.3 (3.8)	8.9 (4.2)	0.000
Experienced sleep disturbances, n (%)*	268 (28.6%)	302 (34.5%)	0.007
Reported substantial fatigue, n (%)*^ψ^	321 (34.3%)	443 (50.6%)	0.000

^*^Significant Chi-square or *t* test *p* value using cutoff of 0.05

^ψ^Significant Chi-square or *t* test *p* value using Bonferroni-adjusted cutoff of 0.002

### Prevalence of distress and factors associated with distress

Approximately 36% (*n* = 311) of the sample suffered from serious distress (13 or higher Kessler-6 score). When Kessler scores were further categorized into four levels, 49.0% (*n* = 429) reported high levels of distress (11 or higher Kessler-6 score). In addition, 50.6% (*n* = 443) of practitioners reported considerable exhaustion; approximately 47% (*n* = 411) experienced at least one burnout symptom; and 34.5% (*n* = 302) reported having sleep issues (trouble falling asleep or staying up at least half the night). Of the 34.5% reporting sleep-related issues, 85.4% (*n* = 258) experienced problems functioning during the day because of these issues.

Bivariate associations between reported levels of distress and various sociodemographic and occupational factors are presented in Table 2. Being younger or female, having fewer years of experience, experiencing a high workload, and reporting dissatisfaction at work were associated with higher levels of distress. Conversely, having effective safety measures in the workplace, receipt of adequate training in the use of PPEs, and having a healthy working environment, were significantly associated with lower levels of distress.

**Table 2:** Demographic, professional and workplace characteristics across distress levels in Jordanian healthcare practitioners experiencing a COVID-19 wave (n = 876). Row total percentages presented

	No distress (n = 13)	Low distress (n = 160)	Moderate distress (n = 274)	High distress (n = 429)	*p* value
Age (mean)* ^ψ^	39.9	35.7	34.1	31.6	0.000
Male*^ψ^	10 (23.1%)	86 (53.8%)	114 (41.6%)	155 (36.1%)	0.000
Live with spouse*	10 (76.9%)	115 (71.9%)	180 (65.7%)	245 (57.1%)	0.003
Have children*	9 (69.2%)	104 (65.0%)	151 (55.1%)	208 (48.5%)	0.002
Live with old people	5 (38.5%)	63 (39.4%)	137 (50.0%)	213 (49.7%)	0.106
Live with young people*	11 (84.6%)	133 (83.1%)	194 (70.8%)	312 (72.7%)	0.023
Education level					0.080
Diploma or less	1 (7.7%)	23 (14.4%)	28 (10.2%)	42 (9.8%)	
Bachelor degree	7 (53.9%)	100 (62.5%)	201 (73.4%)	315 (73.4%)	
Masters, PhD	5 (38.5%)	37 (23.1%)	45 (16.4%)	72 (16.8%)	
Occupation*					
Nurses and technicians	7 (53.9%)	107 (66.9%)	201 (73.4%)	304 (70.9%)	0.003
Specialists and dentists	4 (30.8%)	33 (20.6%)	31 (11.3%)	41 (9.6%)	
GPs and residents	1 (7.7%)	4 (2.5%)	19 (6.9%)	42 (9.8%)	
Pharmacists	1 (7.7%)	16 (10.0%)	23 (8.4%)	42 (9.8%)	
Years of experience in the field (mean)*^ψ^	16	11.8	10.1	8.5	0.000
Site of work					
Hospital ICU & ER, RTU *n* (%)	2 (16.7%)	36 (22.6%)	80 (30.0%)	143 (34.1%)	0.145
Hospital medical departments, *n* (%)	9 (75.0%)	97 (61.0%)	154 (57.7%)	226 (53.8%)	
Other sites, *n* (%)	1 (8.3%)	26 (16.4%)	33 (12.4%)	51 (12.1%)	
Type of institution					
Specialized hospital (cancer)	6 (46.2%)	56 (35.2%)	89 (32.6%)	169 (39.8%)	0.180
Non-cancer/general hospital (government, private or academic)	7 (53.9%)	96 (60.4%)	175 (64.1%)	250 (58.8%)	
Community pharmacy	0 (0.0%)	7 (4.4%)	9 (3.3%)	6 (1.4%)	
Exposed to potential COVID patients in line of work, yes (versus no)	12 (92.3%)	134 (83.8%)	243 (88.7%)	392 (91.4%)	0.065
Work in a COVID-19 specialized ward*	6 (46.2%)	47 (29.4%)	111 (40.5%)	198 (46.2%)	0.003
Experienced a high workload during past 30 days, yes (versus no)*^ψ^	3 (23.1%)	54 (33.8%)	144 (52.6%)	295 (68.8%)	0.000
Was satisfied at work (agree, relative to all other responses)* ^ψ^	12 (92.3%)	139 (86.9%)	200 (73.3%)	226 (52.7%)	0.000
Agreed that co-workers could be relied on to do their jobs well*	7 (53.9%)	100 (62.5%)	140 (51.3%)	204 (47.6%)	0.015
Agreed that peers could openly talk about what was and wasn't working*^ψ^	10 (76.9%)	130 (81.3%)	176 (64.5%)	244 (56.9%)	0.000
Agreed that place of work implemented effective safety measures*^ψ^	10 (76.9%)	116 (72.5%)	171 (62.4%)	218 (50.8%)	0.000
Agreed that sufficient training was provided for use of personal protective equipment*^ψ^	10 (76.9%)	106 (66.3%)	147 (53.7%)	203 (47.3%)	0.000
Availability of full PPE, *n* (%)*	6 (46.2%)	77 (48.1%)	114 (41.6%)	142 (33.1%)	0.005
Reported at least one symptom of Burnout, n (%)*^ψ^	0 (0%)	16 (10.0%)	88 (32.2%)	307 (71.8%)	0.000
Anxiety, past 7 day raw score, mean (SD)* ^ψ^	4.46 (0.78)	7.0 (2.4)	9.33 (2.8)	13.9 (3.21)	0.000
Depression, past 7 day raw score, mean (SD)* ^ψ^	4.60 (2.22)	5.0 (1.5)	6.61 (2.33)	12.0 (3.65)	0.000
Experienced sleep disturbances, n (%)*^ψ^	1 (7.7%)	12 (7.5%)	36 (13.1%)	253 (59.0%)	0.000
Reported substantial fatigue, n (%)*^ψ^	0 (0%)	25 (15.6%)	96 (35.0%)	322 (75.1%)	0.000

^*^Significant *χ*^2^ or ANOVA *p* value using cutoff of 0.05

^ψ^Significant *χ*^2^ or ANOVA *p* value using Bonferroni-adjusted cutoff of 0.002

When distress levels were analyzed using a multivariable ordinal logistic regression, the following variables were found to be significantly associated with a higher of odds of being in greater distress categories (Table 3): reporting burnout, experiencing sleep disturbances, and being fatigued. Specific fears and coping mechanisms that also were related to higher distress levels included having fatalistic fears, and having fears related to workload. Namely, the odds of being in a higher distress level were 59%, and 82% higher for every unit increase in average factor scores reflecting fatalistic fears and workload-related fears. Conversely, being male, reporting satisfaction at work, and using positive coping practices, were associated with significantly lower odds of being in distress. Specifically, men and HCPs reporting satisfaction at work were approximately 50% less likely to be in the greater distress categories; and the odds of being in a higher distress level decreased by 50% for every unit increase in average factor scores reflecting positive coping practices (such as having a positive attitude, talking to others, using prayer or spiritual mechanisms).

**Table 3:** Multivariable ordinal logistic regression examining the association between demographic, psychological and professional characteristics on distress level in a sample of Jordanian healthcare practitioners

	Odds Ratio	*p* value	95% confidence interval	
Male (reference female)*^ψ^	0.52	0.000	0.36	0.75
Age in years	0.98	0.146	0.96	1.01
Married (reference: unmarried)	1.00	0.999	0.64	1.57
Live with young (reference: those who do not)*	1.56	0.041	1.02	2.40
Live with older adults (reference: those who do not)	1.29	0.151	0.91	1.81
Profession (reference: nurses & technicians)				
Pharmacists	1.73	0.229	0.71	4.21
Specialists/dentists	1.61	0.134	0.86	3.00
GPs and residents	1.99	0.079	0.92	4.28
Educational level (reference: Bachelors)				
Diploma or less	1.42	0.208	0.82	2.43
Masters, PhD	0.95	0.836	0.57	1.58
Type of institution (reference: non-cancer/general hospital)				
Community pharmacies	0.88	0.818	0.29	2.67
Tertiary cancer center	1.18	0.419	0.79	1.76
Ward (reference: ICU/ER)				
Other medical wards	0.94	0.773	0.63	1.41
Other sites	0.96	0.923	0.45	2.05
Work directly with COVID patients*	1.57	0.033	1.04	2.37
Agreed that they were satisfied with work (reference: those who disagreed or were neutral to the statement)*^ψ^	0.50	0.001	0.33	0.75
Experienced higher workload ( Reference: those who reported reasonable, between reasonable and calm or calm)	1.09	0.655	0.76	1.56
Reported at least one symptom of burnout (reference: reported no symptoms of burnout)*^ψ^	2.99	0.000	2.02	4.42
Agreed that place of work implemented effective safety measures (reference: those who disagreed or were neutral)	0.82	0.316	0.55	1.21
Had [quite a bit, very much] fatigue (reference: those who reported some or none)*^ψ^	2.40	0.000	1.61	3.56
Experienced [quite a bit, very much] sleep disturbances (reference: those who reported some or none)*^ψ^	4.35	0.000	2.73	6.93
Fears related to respondents’ families (mean value)	1.08	0.589	0.81	1.44
Fears related to the respondent becoming infected (mean value)	0.99	0.942	0.74	1.32
Fatalistic fears (mean value)*^ψ^	1.59	0.001	1.22	2.07
Fear of quarantine (mean value)	1.08	0.541	0.84	1.38
Fears related to workload (mean value)*^ψ^	1.82	0.000	1.39	2.39
Monetary fears (mean value)	1.16	0.153	0.95	1.42
Coping using positive practices (mean value)*^ψ^	0.51	0.000	0.37	0.70
Coping by seeking COVID-related information and controlling risk of infection (mean value)	0.99	0.971	0.71	1.39
Coping using denial, avoidance (mean value)*	1.48	0.008	1.11	1.97
Reported PPEs available	1.36	0.102	0.94	1.98

^*^Significant *p* value using cutoff of 0.05

^ψ^Significant *p* value using Bonferroni-adjusted cutoff of 0.002

In both cross-sectional studies, similar trends were observed with regards to variables impacting distress, with the exception of certain demographic and professional characteristics, such as older age (older age was significantly associated with lower distress in the multivariable analysis of the first study period), living with younger people (this was associated with greater distress only in period 2), being a pharmacist (this was associated with greater distress in period 1), and type of institution (working in a tertiary cancer center was associated with greater distress in period 1).

### Coping mechanisms

Coping mechanisms were included in the multivariable regression to evaluate their association with distress levels. However, we also were interested—from a descriptive perspective—in deciphering which coping strategies tended to be used the most. These included following strict personal protective measures (e.g., mask, gown, hand washing etc.); keeping separate clothes for work/used disposable scrubs to minimize transmission; considering every patient admitted to the hospital as COVID-19; using full protective gear even if a patient was COVID-19 negative; reading about COVID-19; avoiding going out in public places to minimize exposure; and employing prayer or spiritual coping strategies were employed “most” or “all” of the time in 75% or more of respondents.

### Importance of select factors for HCPs’ work

With regards to degree of importance of a selection of items for HCPs’ work, those cited most frequently as “important” or “most important” were: having adequate PPE supplies (91.7% of respondents), coverage of treatment if the HCP was infected and required intensive care (89.8%), and family support (88.1%). The least cited factor in terms of importance was poor media representation (58.4%).

## Discussion

Our study sought to compare a sample of Jordanian HCPs experiencing a sharp COVID-19 wave with a similar sample of HCPs during a period of low COVID-19 caseloads, to evaluate differences in distress level and factors impacting distress in these two distinct phases of the pandemic. Despite HCPs exhibiting high distress levels even when COVID-19 rates were low, we found that levels of distress were considerably higher in the current sample experiencing a sharp COVID-19 wave. Furthermore, approximately half the respondents (versus roughly a third in the first sample) suffered from severe distress, and mean depression and anxiety scores were roughly 21% higher. Variables that continued to be significantly associated with being in higher distress regardless of COVID-19 burden included being female, not being satisfied at work, suffering from burnout and sleep problems or fatigue, and harboring fatalistic fears about COVID-19 and fears related to workload. Other notable results in our study include the persisting proportion of respondents who did not have full PPE available to them. Overall, 45% of respondents did not have full PPE.

Some of our findings are comparable to what other studies of Jordanian HCPs have reported. For example, others have also found that female Jordanian HCPs report significantly more negative mental health outcomes relative to males [15, 17]; and substantial burnout has been reported by Alrawashdeh et al. [15] Variation in levels of anxiety and distress across other studies conducted in Jordan are likely to be the effect of using different measurement tools across studies as well as substantial differences in sample characteristics [14, 18].

Our results also shed light on a previously unexplored aspect, namely, coping mechanisms used by Jordanian HCPs, and what HCPs considered important factors or conditions related to their work and profession. Employing positive coping mechanisms (e.g., positive attitude, talking to others), was associated with being in a lower distress category. Furthermore, the availability of PPE, having medical coverage in the event of becoming ill, and family support, were key factors of importance echoed by respondents.

In line with our previous findings [16], the strong associations observed between distress and HCP burnout, fatigue and sleep problems underscore the importance of Jordanian medical institutions implementing employee wellness programs. The observation of a protective effect for using constructive coping mechanisms also highlights an opportunity to educate HCPs with regards to critical topics, such as coping, resilience, and stress management, and the value of cognitive restructuring in respondents who tend to harbor fatalistic fears. Availing educational opportunities in this area can both enhance HCPs’ ability to navigate challenging work environments while also improving HCPs’ satisfaction at work.

Minor differences were observed between the two samples with regards to profession. Although Pharmacists reported high levels of distress (relative to other practitioners) in the first cross-sectional survey, this was not observed during period 2, likely due to the unique and tiring situation pharmacists found themselves in during the lockdown period (period 1) in Jordan. During that time, pharmacists’ workloads were unusually high.

Our study has some limitations. Although our cross-sectional samples were comparable in terms of demographic characteristics, differences in distress levels between the two pandemic periods could have been better ascertained using a longitudinal study design. It is, therefore, arguable that, had we repeated the survey using the same sample, a different result would have been observed. At the time of the study, doing so was not possible. Nevertheless, levels of distress in the second cross-sectional sample were not significantly different when we compared distress in a small proportion of respondents who had completed both surveys (*n* = 232) with distress in the remaining respondents (who had not filled out the survey in period 1). Furthermore, although we accessed a relatively large sample of HCPs, our sample was an opportunistic one, which arguably could impact the sample’s external validity. We also explored coping strategies as well as motivators and barriers to work in a quantitative manner. Thus, in-depth perspectives were not captured. Such an in-depth analysis would have required qualitative methods to reveal the complex realities and nuanced lived experiences of our sample.

Worldwide, there are limited published studies on HCPs to illustrate COVID-19-associated changes in mental health over time as pandemic waves ebb and flow. The few studies conducted have varied in their findings. For example, some have reported greater levels of depression and other negative mental health outcomes during COVID-19 outbreaks, and improvements in mental health during periods of stability [41]. Others have reported persisting if not worse findings in periods of stability [42]. Th’ng et al. in a longitudinal study, demonstrated that physicians in particular reported worsening depression with time [43]. Similar to our results, Th’ng et al. reported higher odds of depression and anxiety among HCPs who had infection-related and workload-related concerns, and lower odds of depression and anxiety among HCPs with better perception about their working environment. A four-wave longitudinal study carried out in Japan showed that HCPs continuously experienced high psychological distress during the study period, even when caseloads were lower [44]. Further studies with the aim of capturing changes at different and prolonged timepoints have been launched and are ongoing [45–47].

To date, no longitudinal studies have been conducted in Jordan. To the best of our knowledge, this is the first study that has attempted to demonstrate change in distress in Jordanian HCPs in relation to the changing COVID-19 pandemic. The main strength of this study lies in the repeated cross-sectional design that we used to study differences in mental health symptoms among comparable groups (in terms of sociodemographic characteristics) of HCPs during two different phases of the pandemic, using a consistent methodology.

## Conclusions

Our study demonstrated differences in levels of distress in Jordanian HCPs between an early pandemic period and a period when high caseloads occurred. Our results indicate that HCPs’ levels of fear, distress, anxiety, depression, worsening sleep quality and fatigue increased dramatically despite already being predisposed to the possibility of COVID-19 spread. Moreover, specific sociodemographic, attitudinal and occupational factors continued to significantly influence psychological distress. Specifically, the strong negative associations observed between distress and HCP burnout, fatigue and sleep problems, and the continuing protective effect of workplace satisfaction, underscore the importance of Jordanian medical institutions implementing employee wellness programs and employing strategies to improve work environments.

## Supplementary Information

**Additional file 1:** Mitigating psychological distress in healthcare workers as COVID-19 waves ensue: a repeated cross-sectional study from Jordan

## Data Availability

Data cannot be shared publicly because of institutional regulations. Data requests are reviewed and approved by the Institutional Review Board at King Hussein Cancer Center (contact Ms. Linda Kateb, at IRBOffice@KHCC.JO). For researchers who meet the criteria for access to confidential data, data can then be shared.

## Abbreviations

AAHRPP: Association for the Accreditation of Human Research Protection Programs
COVID-19: Coronavirus disease 2019
HCPs: Healthcare practitioners
PPE: Personal protective equipment
PROMIS: Patient-Reported Outcomes Measurement Information System
SARS: Severe acute respiratory syndrome
SARS-CoV-2: Severe acute respiratory syndrome coronavirus 2
