# Supplementary material for: Mitigating psychological distress in healthcare workers as COVID-19 waves ensue: a repeated cross-sectional study from Jordan
Source: Hum Resour Health. 2022 Apr 11;20:32. doi: 10.1186/s12960-022-00728-x (PMC8995692; doi:10.1186/s12960-022-00728-x)
Supplement: Supplementary file 1 — Additional file 1: Mitigating psychological distress in healthcare workers as COVID-19 waves ensue: a repeated cross-sectional study from Jordan [file 12960_2022_728_MOESM1_ESM.docx]

Exploratory factor analyses were conducted to decipher if the numerous variables used to measure fear related to Covid-19 and coping strategies employed were represented by an underlying set of reduced factors. (1-3)

**Covid-related fears**

Fear items were first assess to ensure they were appropriately distributed for an exploratory factor analysis (EFA). The KMO value indicated that the sample was factorable and homogeneity of variance was confirmed by Bartlett’s test of sphericity. Parallel analysis (PA) was performed to determine number of factors to be retained from factor analysis. Unlike our first survey,(4) where no decipherable factors were obtained, in our current analysis, 21-items were reduced to six factors which we labeled as follows: Fears related to respondents’ families; fears related to the respondent becoming infected; fatalistic fears about the virus being out of control and thoughts about death; quarantine fears; fears related to nature of work; and monetary fears. Two items were dropped due to failing to load on any factors: fear of infecting colleagues, and fears related to respondents other health concerns. Within factors, the internal consistency of items was consistently 0.7 or higher.

Average scores were generated for the six factors and used in subsequent regression analyses.(5) The items contributing to each factor are summarized below.

| **Fear item** | **Factor loading on** |
| --- | --- |
| Covid-19 makes me feel that life is threatened | Fatalistic fear |
| Covid-19 makes me feel that I have lost control of life | Fatalistic fear |
| Covid-19 makes me think about death | Fatalistic fear |
| Covid-19 makes me feel that the virus will get out of control–spread continuously | Fatalistic fear |
| Covid-19 makes me fear that I will be infected | Fear of being infected |
| Covid-19 makes me suspect whether I have been infected or not | Fear of being infected |
| Covid-19 makes me feel that the virus is very close to me, (and the virus may) invade my body anytime | Fear of being infected |
| Covid-19 makes me feel very unsafe | Fear of being infected |
| Covid-19 makes me fear that I will infect my family members at home | Fears about family |
| Covid-19 makes me worry if my family will be infected | Fears about family |
| Covid-19 makes me think about myself–families–colleagues getting infected | Fears about family |
| Covid-19 makes me worry about other health problems regarding my family members | Fears about family |
| Covid-19 makes me feel distressed because of the upsurge in workload | Fears related to nature of work |
| Covid-19 makes me feel I am being discriminated against by others | Fears related to nature of work |
| Covid-19 makes me worry if my family or friends will keep a distance from me (I will be alienated) due to my job duties | Fears related to nature of work |
| Covid-19 makes me worry if I will be assigned to Covid-19-infected wards | Fears related to nature of work |
| Covid-19 makes me worry about my personal financial matters as a result of the outbreak | Monetary fears |
| Covid-19 makes me worry about the financial matters of others who mean to me, as a result of the outbreak | Monetary fears |
| Covid-19 makes me fear being quarantined at home | Quarantine fears |
| Covid-19 makes me fear being quarantined in a quarantine site (field hospital or hotel) | Quarantine fears |
| Covid-19 makes me fear my family being quarantined | Quarantine fears |

**Coping strategies used by healthcare practitioners**

We applied the same EFA and PA method used for determining the source of fears to analyze the factorial structure and extract the main components that underlie different coping strategies adopted by the HCWs. As with our approach with Covid-related fears, we confirmed that the data were factorable (favorable KMO value and confirmed homogeneity of variance). 14-items were reduced to three factors which we labeled as follows: coping using positive practices; coping by seeking Covid-related information and controlling risk of infection; and coping using denial, avoidance, crying, or negative reactions. One item was dropped due to failing to load on any factors: seeking psychological help. Within factors, the internal consistency of items was consistently 0.7 or higher.

Average scores were generated for the three factors and used in subsequent regression analyses.(5) The items contributing to each factor are summarized below.

| **Strategy used by staff** | **Factor loading on** |
| --- | --- |
| Follow strict personal protective measures (e.g., mask, gown, hand washing etc.) | Coping by seeking Covid-related information and controlling risk of infection |
| Keep separate clothes for work/used disposable scrubs provided by Hospital to minimize transmission | Coping by seeking Covid-related information and controlling risk of infection |
| Consider every patient admitted to the hospital as having SARS-CoV-2 infection and using full protective gear even if patient was SARS-CoV-2 negative | Coping by seeking Covid-related information and controlling risk of infection |
| Read about SARS-CoV-2, its prevention and mechanism of transmission | Coping by seeking Covid-related information and controlling risk of infection |
| Avoid going out in public places to minimize exposure from SARS-CoV-2 | Coping by seeking Covid-related information and controlling risk of infection |
| Perform relaxation activities, e.g., involved in sports, exercise etc. | Coping using positive practices |
| Prayer, spirituality | Coping using positive practices |
| Chat with family and friends to relieve stress and obtain support | Coping using positive practices |
| Talk to self and motivate self to face the SARS-CoV-2 outbreak with positive attitude | Coping using positive practices |
| Try to be busy in activities that would keep your mind away from SARS-CoV-2 | Coping using denial, avoidance, crying, negative reactions |
| Avoid doing overtime to reduce exposure to SARS-CoV-2 patients in hospital | Coping using denial, avoidance, crying, negative reactions |
| Avoid media news about SARS-CoV-2 and related fatalities | Coping using denial, avoidance, crying, negative reactions |
| Vent by crying, screaming etc. | Coping using denial, avoidance, crying, negative reactions |
| Vent through bad habits | Coping using denial, avoidance, crying, negative reactions |

1. Pituch, K.A., & Stevens, J.P. (2015). Applied Multivariate Statistics for the Social Sciences: Analyses with SAS and IBM’s SPSS, Sixth Edition (6th ed.). Routledge. .

2. Watkins, M. W. (2021). A Step-by-Step Guide to Exploratory Factor Analysis with Stata. Routledge. 2021.

3. Goretzko D, Pham TTH, Bühner M. Exploratory factor analysis: Current use, methodological developments and recommendations for good practice. Current Psychology. 2019;40(7):3510-21.10.1007/s12144-019-00300-2

4. Hawari FI, Obeidat NA, Dodin YI, Albtoosh AS, Manasrah RM, Alaqeel IO, et al. The inevitability of Covid-19 related distress among healthcare workers: Findings from a low caseload country under lockdown. PLoS One. 2021;16(4):e0248741.10.1371/journal.pone.0248741

5. Distefano C., Zhu, M. & Mindrila, D. (2008). Understanding and Using Factor Scores: Considerations for the Applied Researcher. Pract. Assess. Res. Eval.. 14. .10.7275/da8t-4g52
